# 161. The Agency for Healthcare Research and Quality (AHRQ) Safety Program for Improving Antibiotic Use: Antibiotic Stewardship Intervention in 389 United States Ambulatory Practices during the COVID-19 Pandemic

**DOI:** 10.1093/ofid/ofab466.161

**Published:** 2021-12-04

**Authors:** Sara C Keller, Tania M Caballero, Pranita Tamma, Pranita Tamma, Melissa A Miller, Prashila Dullabh, Roy Ahn, Savyasachi V Shah, Yue Gao, Kathleen Speck, Sara E Cosgrove, Sara E Cosgrove, Jeffrey A Linder

**Affiliations:** 1 Johns Hopkins University School of Medicine, Baltimore, MD; 2 Johns Hopkins, Baltimore, MD; 3 Agency for Healthcare Research and Quality, Rockville, Maryland; 4 NORC at the University of Chicago, Bethesda, Maryland; 5 NORC, Chicago, Illinois; 6 Northwestern University Feinberg School of Medicine, Chicago, IL

## Abstract

**Background:**

The AHRQ Safety Program for Improving Antibiotic Use aimed to improve antibiotic use by engaging clinicians and staff to incorporate antibiotic stewardship (AS) into practice culture, communication, and decision making. We report on changes in visits and antibiotic prescribing in AHRQ Safety Program ambulatory practices during the COVID-19 pandemic.

**Methods:**

The Safety Program used webinars, audio presentations, educational tools, and office hours to engage clinician champions and staff leaders to: (a) address attitudes and culture that pose challenges to judicious antibiotic prescribing and (b) incorporate best practices for the management of common infections into their workflow using the Four Moments of Antibiotic Decision Making framework. Total visits (in-person and virtual), acute respiratory infection (ARI) visits, and antibiotic prescribing data were collected. Using linear mixed models to account for random effects of participating practices and repeated measurements of outcomes within practices over time, data from the pre-intervention period (September-November 2019) and the Ambulatory Care Safety Program (December 2019-November 2020) were compared.

**Results:**

Of 467 practices enrolled, 389 (83%) completed the program, including 162 primary care practices (42%; 23 [6%] pediatric), 160 urgent care practices (41%; 40 [10%] pediatric), and 49 federally-supported practices (13%). 292 practices submitted complete data for analysis, including 6,590,485 visits. Visits/practice-month declined March-May 2020 but gradually returned to baseline by program end (Figure 1). Total antibiotic prescribing declined by 9 prescriptions/100 visits (95% CI: -10 to -8). ARI visits/practice-month declined significantly in March-May 2020, then increased but remained below baseline by program end (Figure 2). ARI-related antibiotic prescriptions decreased by 15/100 ARI visits by program end (95% CI: -17 to -12). The greatest reduction was in penicillin class prescriptions with a reduction of 7/100 ARI visits by program end (95% CI: -9 to -6).

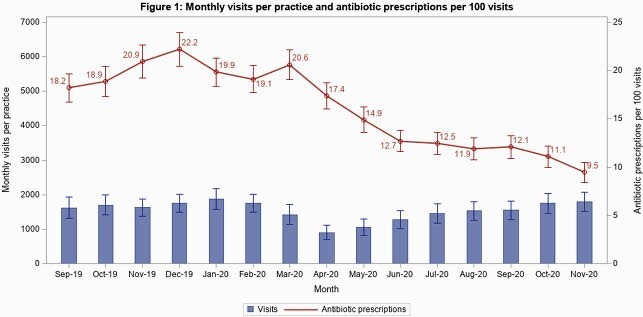

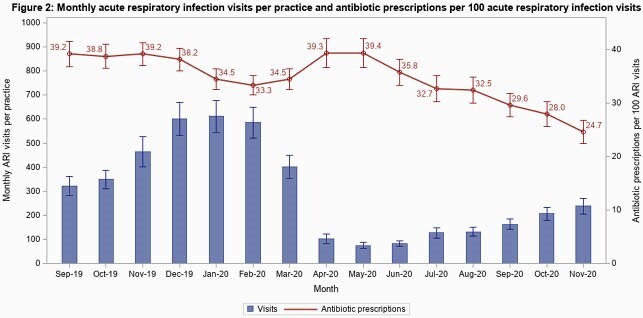

**Conclusion:**

During the COVID-19 pandemic, a national ambulatory AS program was associated with declines in overall and ARI-related antibiotic prescribing.

**Disclosures:**

**Pranita Tamma, MD, MHS**, Nothing to disclose **Sara E. Cosgrove, MD, MS**, Basilea (Individual(s) Involved: Self): Consultant **Jeffrey A. Linder, MD, MPH, FACP**, **Amgen** (Shareholder)**Biogen** (Shareholder)**Eli Lilly** (Shareholder)

